# Chronic kidney disease: susceptibility in a representative population-based sample

**DOI:** 10.11606/S1518-8787.2018052017410

**Published:** 2018-07-17

**Authors:** Chislene Pereira Vanelli, Rogério Baumgratz de Paula, Mônica Barros Costa, Marcus Gomes Bastos, Layla de Souza Pires Miranda, Fernando Antonio Basile Colugnati

**Affiliations:** I Universidade Federal de Juiz de Fora. Faculdade de Medicina. Programa de Pós-Graduação em Saúde. Juiz de Fora, MG, Brasil; II Universidade Federal de Juiz de Fora. Faculdade de Medicina. Juiz de Fora, MG, Brasil

**Keywords:** Renal Insufficiency, Chronic, Diagnosis, Prevalence, Risk Factors, Early Diagnosis, Insuficiência Renal Crônica, Diagnóstico, Prevalência, Fatores de Risco, Diagnóstico Precoce

## Abstract

Chronic kidney disease has high morbidity and mortality. In order to track the disease, we conducted a population-based study in a medium-sized city in Southeastern Brazil. Based on instrument SCreening for Occult REnal Disease (SCORED), we evaluated 1,016 individuals with mean age of 44 (SD = 13.2) years. High blood pressure and diabetes mellitus, major causes of chronic kidney disease, were reported by 34.7% and 10.5% of the individuals, respectively. In addition, 31.3% of the sample presented increased risk for the disease, thus leading to a prevalence estimate of 5.4%. A simple screening method allowed the early detection of a population at risk for chronic kidney disease.

## RESUMO

A doença renal crônica apresenta elevada morbimortalidade. Com o objetivo de rastrear a doença, realizou-se estudo de base populacional em cidade de porte médio do sudeste brasileiro. Com base no instrumento *SCreening for Occult REnal Disease* (SCORED) foram avaliados 1.016 indivíduos com média de idade 44 (DP = 13,2) anos. Hipertensão arterial e diabetes mellitus, principais causas de doença renal crônica, foram relatados por 34,7% e 10,5%, respectivamente. Além disso, 31,3% da amostra apresentou risco aumentado para a doença, levando a uma estimativa de prevalência de 5,4%. Um método simples para rastreio permitiu a detecção precoce de população de risco para doença renal crônica.

## INTRODUCTION

In Brazil, it is estimated that more than two million individuals have some degree of renal dysfunction, and 100,000 of them are in renal replacement therapy, which generates expenditures of approximately 10% of the health budget. Among the main causes of chronic kidney disease (CKD), we can mention diabetes mellitus (DM) and high blood pressure (HBP), which are prevalent chronic conditions and frequently underdiagnosed in the country^1^.

Thus, the early detection of CKD is fundamental, since it allows the implementation of measures that act on the natural evolution of the disease, which decrease the occurrence of complications and the need for renal replacement therapy^2^.

In Brazil, there is a lack of population-based studies focused on the prevalence of CKD, which allow the tracing of the population profile and subsidize measures to delay the evolution of the disease.

This study aimed to screen for CKD, based on the self-report of the presence of factors associated with the disease, in a population-based sample representative of a medium-sized city in the Southeast region of Brazil.

## METHODS

This is a population-based, cross-sectional study carried out in the city of Juiz de Fora, State of Minas Gerais, Brazil, with data collected from June/2014 to April/2016. We used a three-stage sampling of households, stratified by the seven administrative regions – center, east, west, north, northeast, south, and southeast – of the primary health care units (PHCU), using census tracts as primary sample units. In the first stage, we selected the conglomerates (census tracts), with probability proportional to size and systematic selection, with size according to the population living in permanent private households. In the second stage, we selected a fixed number of households in each conglomerate, and we systematically selected the households from the list of addresses provided by the Brazilian Institute of Geography and Statistics (IBGE, 2010). In the third stage, we randomly selected the residents aged ≥ 18 years.

A total of 4,800 households were visited, and we randomly selected 1,032 individuals who met the inclusion criteria and signed the informed consent. The sample size provided prevalence estimates with a sampling error of five percentage points, above or below, with a 95% confidence level. For the screening of CKD, we used the SCreening for Occult REnal Disease (SCORED)^4^ instrument, which is a questionnaire with nine questions with different weights that aims to predict the chance of a particular individual presenting CKD. According to this instrument, an individual is more likely to have CKD if they score four or more points in the questionnaire.

The study was authorized by the Municipal Health Department of Juiz de Fora, Minas Gerais, and approved by the Human Research Ethics Committee of the University Hospital of the Universidade Federal de Juiz de Fora (Protocol 133.399). The collected data were stored on the RedCap^®^ platform, with subsequent analysis using the Stata^®^ software, version 13.1.

## RESULTS

From 1,032 interviews, we used the SCORED instrument in 1,016 participants. The mean age of the responders was 44 (SD = 13.2) years, and 722 (71.1%) were females. Based on self-report, 34.7% of the individuals reported previous diagnosis of HBP and 10.5% reported a diagnosis of DM.

The table presents the variables included in the SCORED questionnaire. We observed that 318 (31.3%) individuals had a 20% chance of presenting CKD, that is, they reached a score equal to or greater than four in the instrument.

**Table:** Screening of chronic kidney disease in adults of a medium-sized city in the Southeast region of Brazil.

Parameters	n	%
Age (years)		
50–59	290	28.5
60–69	132	13.0
≥ 70	0	0
Female	722	71.1
Anemia	174	17.1
High blood pressure	352	34.7
Diabetes mellitus	107	10.5
Cardiovascular disease	180	17.7
Albuminuria	42	4.4
Score ≥ 4	318	31.3

## DISCUSSION

In this study, with a representative sample of the population of a city in Southeastern Brazil, we observed a high risk for diagnosis of CKD and a high prevalence of DM and HBP, which are recognized as the main causes of CKD worldwide.

We highlight that, in a relatively young population, in which 58.5% of the respondents were younger than 50 years, we detected a high risk for CKD, which shows the need to screen for chronic health conditions even in asymptomatic individuals. It is important to note that, considering the high number of young individuals in the study population, the presence of CKD may have been underestimated, since approximately 600 individuals did not score on the screening instrument, based on the “age” criterion, since only individuals aged ≥ 50 years can have a score on SCORED.

In relation to the high prevalence of HBP and DM, both conditions present an asymptomatic evolution, hindering early diagnosis and, consequently, screening for CKD. The prevalence rate of self-reported HBP in the studied sample, consistent with data from the world literature, is different from the findings of the National Health Survey (PNS)^3^ of 2013, in which a prevalence of 21.4% was observed in a representative sample of the Brazilian population. Similarly, the prevalence of DM was higher than the national estimates of the PNS of 2013, in which 6.2% of the participants reported a previous diagnosis of the disease. In contrast, according to the International Diabetes Federation of 2015, the prevalence of DM in the adult population worldwide was 8.8% and the prevalence at the national level was 10.4%, which are similar to the data in this study.

Another relevant finding was the report of a previous diagnosis of cardiovascular disease (CVD) in approximately 20% of the sample, regardless of the age group. Cardiovascular disease mortality is the main cause of death in the population with CKD, which reinforces the need for screening for chronic health conditions in our population.

In parallel, the presence of albuminuria, a key element in the stratification of CKD because of its diagnostic and prognostic importance, was reported in a small number of individuals studied. Such a finding suggests that, this examination has been underused in screening in the primary health care, as observed by other authors^2^.

Based on the above, the use of risk assessment instruments for CKD, such as the SCORED, is very useful in the general population. According to the validation properties of SCORED in Brazil^4^, a positive predictive value of 14% and sensitivity of 80% were found, and considering the finding in our study – 31.3% of tests with a score equal or superior to four – we can estimate a prevalence of CKD in approximately 5.4% of the sample evaluated^a^.

The prevention and delay of CKD progression is directly related to early detection, since the best results in CKD care are obtained in individuals who are forwarded early to specialized health care services^5^.

One limitation of this study is the high number of females, which may be related to the greater availability of women in the visited households. In addition, women are predominant in the population of the city evaluated.

Our results showed an increased risk for the diagnosis of CKD in approximately 31% of a relatively young and asymptomatic population. In the context of public health, a simple and easily implemented screening method allowed the early detection of risk for CKD. Thus, we suggest that a similar strategy can be of great value in the elaboration of policies for CKD prevention and surveillance in Brazil.

## Footnotes

a This estimate is made from the conditional probabilities involved, where: Prob(CKD) = PPV × P(SCORED ≥ 4) / Sensitivity
